# Integrative Analysis Reveals Subtype-Specific Regulatory Determinants in Triple Negative Breast Cancer

**DOI:** 10.3390/cancers11040507

**Published:** 2019-04-10

**Authors:** Shujun Huang, Wayne Xu, Pingzhao Hu, Ted M. Lakowski

**Affiliations:** 1 College of Pharmacy, University of Manitoba, Winnipeg, MB R3E 0T5, Canada; huangs12@myumanitoba (S.H.); Wayne.xu@canada.ca (W.X.); 2 Department of Biochemistry and Medical Genetics, University of Manitoba, Winnipeg, MB R3E 0J9, Canada; 3 Research Institute in Oncology and Hematology, University of Manitoba, Winnipeg, MB R3E 0V9, Canada

**Keywords:** breast cancer, regulator, lasso, triple negative, PPARA, FOXM1

## Abstract

Different breast cancer (BC) subtypes have unique gene expression patterns, but their regulatory mechanisms have yet to be fully elucidated. We hypothesized that the top upregulated (Yin) and downregulated (Yang) genes determine the fate of cancer cells. To reveal the regulatory determinants of these Yin and Yang genes in different BC subtypes, we developed a lasso regression model integrating DNA methylation (DM), copy number variation (CNV) and microRNA (miRNA) expression of 391 BC patients, coupled with miRNA–target interactions and transcription factor (TF) binding sites. A total of 25, 20, 15 and 24 key regulators were identified for luminal A, luminal B, Her2-enriched, and triple negative (TN) subtypes, respectively. Many of the 24 TN regulators were found to regulate the PPARA and FOXM1 pathways. The Yin Yang gene expression mean ratio (YMR) and combined risk score (CRS) signatures built with either the targets of or the TN regulators were associated with the BC patients’ survival. Previously, we identified FOXM1 and PPARA as the top Yin and Yang pathways in TN, respectively. These two pathways and their regulators could be further explored experimentally, which might help to identify potential therapeutic targets for TN.

## 1. Introduction

Breast cancer (BC) is one of the most common cancers in the world [1]. In the US, approximately one in eight women (about 12.4%) will develop invasive BC over the course of her lifetime [2]. Despite an overall decline in incidence and mortality, BC remains the second leading cause of cancer-related death among women worldwide [1]. BC can be categorized into four main molecular subtypes, luminal A, luminal B, Her2-enriched and triple negative (TN), depending on the expression of the estrogen receptor (ER), progesterone receptor (PR) and epidermal growth factor receptor 2 (ERBB2, also known as HER2) [3]. TN (also called basal-type) tumors are ER negative, PR negative and HER2 negative. Although the TN subtype is only found in approximate 15% of BC diagnoses, it has been shown to be aggressive, unresponsive to treatment, and ultimately indicative of a poor prognosis [4,5].

Changes in gene expression resulting from alterations in microRNA (miRNA) expression, DNA methylation (DM) and DNA copy number have been implicated in breast carcinogenesis [6]. The role of miRNAs is to regulate target mRNA translation and degradation by binding to the 3′ untranslated region (UTR) [7], and their deregulation is associated with BC initiation and progression [6]. In cancer, DM is perturbed, leading to significant changes in gene expression. For example, aberrant CpG hypermethylation has been reported in multiple genes associated with BC, typically leading to gene silencing [6]. Genomic instability due to copy number variation (CNV) is also associated with altered gene expression in breast carcinogenesis [8]. Transcription factors (TFs) are major regulators of gene expression, and can serve either as transcriptional activators or repressors [9]. High-throughput approaches allow us to investigate a tumor at multiple levels. For example, the Cancer Genome Atlas (TCGA) database contains cancer-related omics profiles that are useful for systems-biology-based investigations of BC. Therefore, research on the regulatory mechanisms of gene expression changes should take the interaction of multiple factors into consideration.

Considering multiple factors regulating gene expression presents a challenge in terms of combining data from different experiments to generate biologically meaningful and experimentally testable models. Interaction-network-based approaches that analyze individual genes and their interactions as well as upstream regulatory mechanisms are an effective approach to overcome this challenge. For example, Xu et al. proposed a method, weighted similarity network fusion (WSNF), to utilize the information in a miRNA-TF-mRNA regulatory network to successfully identify BC subtypes [10]. In addition, this study showed that the top pathways involving the most differentially expressed genes (DEGs) in each of the identified subtypes are different, but they did not further explore the regulatory machinery behind the DEGs. A recent study identified hsa-miR-301a- and SOX10-dependent miRNA-TF-mRNA regulatory circuits in BC by using previously performed expression studies, but subtype information was not included in this study [11].

An integrative strategy using regression models to combine data at multiple levels can also provide insight for gene expression deregulation in cancer. Setty et al. developed a regularized linear regression model in a glioblastoma multiforme (GBM) study using data from the Cancer Genome Atlas (TCGA) [12]. The model was fit to changes in mRNA expression in each GBM tumor sample as the response variable, using a linear combination of the input variables including the CNV, DM, miRNA sequence-based predictions, and TF-binding sites. The model successfully identified various key miRNAs and TFs as either common or subtype-specific drivers of expression changes. The estimated activities of these key regulators were predictive to GBM subtypes and patient survival. Their work took global gene expression changes into account. These studies suggest that similar approaches may be used to explore the regulatory mechanisms in the different subtypes of BC.

Comparing cancer tissue samples with normal tissue has been historically used for discovery of DEGs in cancer; however, few studies have looked at them by way of two opposing groups of genes, the interaction of which would affect cancer progression. In this study, we hypothesized that two opposing effects in BC cancer cells, represented by Yin and Yang genes, determine the outcome of BC [13,14]. Yang genes are expressed more highly in normal breast tissues, while Yin genes are expressed more highly in breast tumors. Assuming that the changes in Yin and Yang gene expression are influenced by variation in copy number, DNA methylation, TF deposition and miRNA–mRNA interaction, we input these variables into a sample-specific lasso regularized linear regression model. The aim of this study was to elucidate the predominant regulators of the Yin and Yang genes in different BC subtypes. We focused our follow-up exploration on the TN subtype, and evaluated the clinical relevance of key miRNA/TF regulators.

## 2. Results

### 2.1. Selection of Yin and Yang Genes

**Figure 1:**
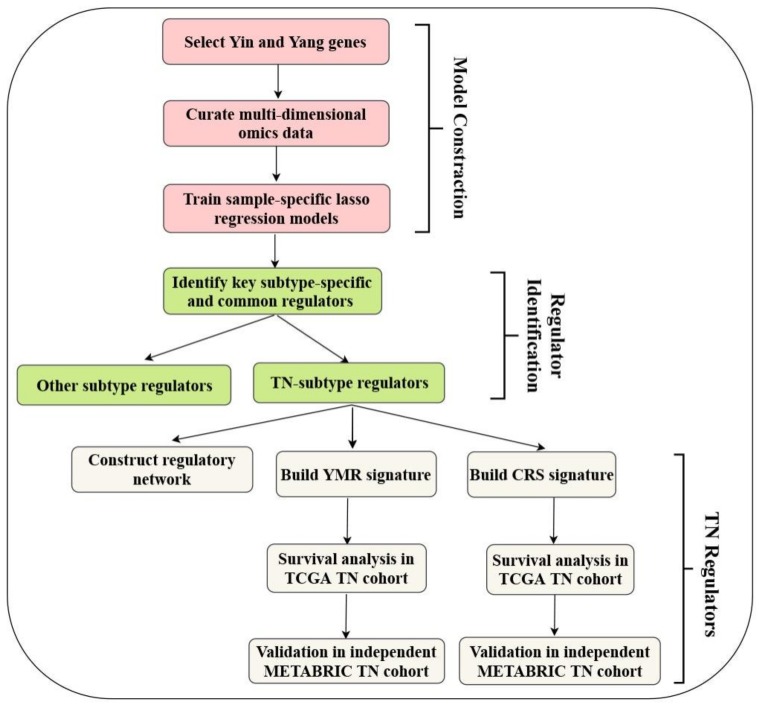
Study design. This study included three major parts. The first was to construct sample-specific models. Key regulators in different breast cancer subtypes were then identified from the models. Finally, we focused our follow-up exploration on the regulators in the TN subtype.

**Figure 2:**
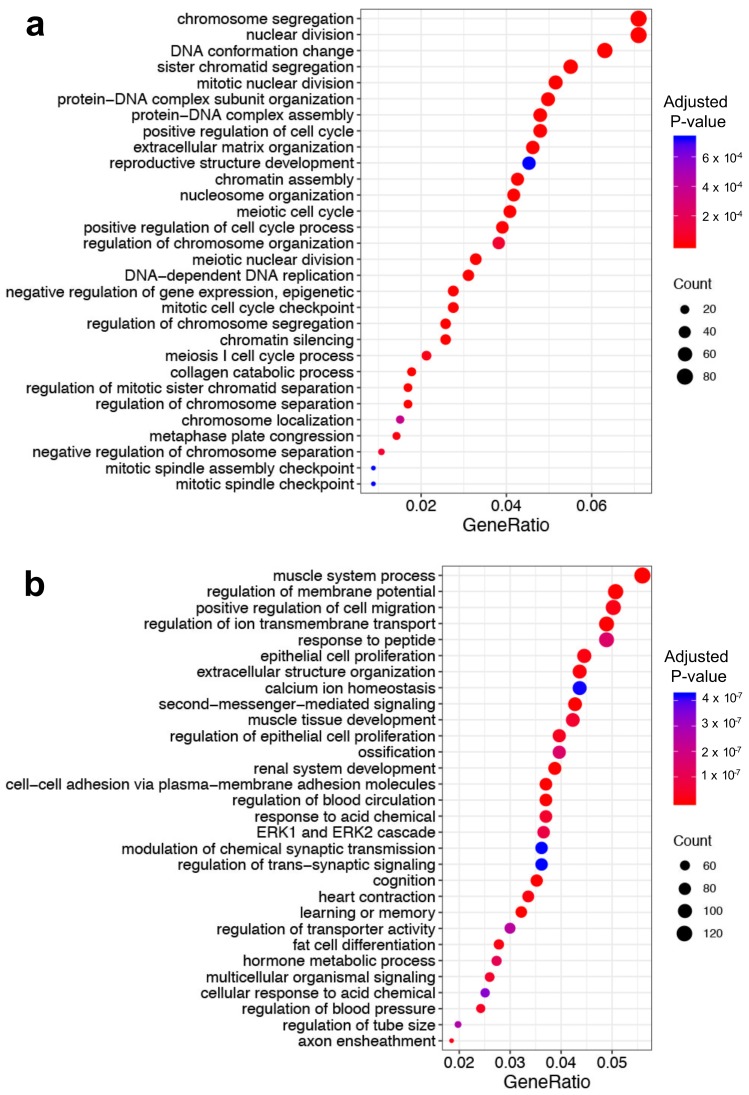
Top 30 over-represented gene ontology (GO) terms in biological process (BP) (GO-BP) terms in the Yin (**a**) and Yang (**b**) genes. “GeneRatio” in axis x is the ratio of the number of the identified Yin or Yang genes included in the corresponding term (“Count”) over the total number of the identified Yin or Yang genes. The y-axis is a description of GO-BP terms.

As shown in the study design (Figure 1), we focused on Yin and Yang gene expression changes instead of global gene expression changes. Based on our Yin Yang hypothesis, “Yang” genes are downregulated DEGs in BC but express more highly in normal breast tissues, while “Yin” genes are upregulated DEGs that express more highly in breast tumors. 4227 of the 17,675 genes we studied were significantly (false discover rate (FDR) < 0.05) differentially expressed between BC and normal tissue samples, with the absolute value of logFC (log2 fold change) not less than 1. Among these DEGs, 1413 were Yin (upregulated) genes, while 2814 were Yang (downregulated) genes. For the 1413 Yin genes, 255 gene ontology (GO) terms in biological process (BP) (GO-BP) were obtained with an adjusted *p*-value < 0.05. The majority are involved in cell proliferation, such as DNA replication (GO:0006260), chromosome segregation (GO:0007059), nuclear division (GO:0000280), cell cycle checkpoint (GO:0000075) and cytokinesis (GO:0000910). The similarities among the 255 GO-BP terms were calculated using the *simplify* function in the clusterProfiler package [15], and highly similar terms (which have similarity higher than 0.7) were removed by keeping one representative term. In the end, 239 terms were obtained. For the 2814 Yang genes, 1155 GO-BP terms were overrepresented with adjusted *p*-value < 0.05. The *simplify* function was also applied to these 1155 terms, and 239 terms were kept in the end. The top two (with the smallest adjusted *p*-value) were muscle system process (GO:0003012) and cell–cell adhesion via plasma membrane adhesion molecules (GO:0098742). When the 239 terms were ordered according to the number of Yang genes that were involved in the GO-BP terms (“Count”), nine terms included more than 100 Yang genes. Muscle system process (GO:0003012) was the top one which included 127; positive regulation of cell migration (GO:0030335) included 114; organic hydroxy compound metabolic process (GO:1901615) included 110, and epithelial cell proliferation (GO:0050673) includes 101 of the 2814 Yang genes. Figure 2 shows the top 30 most significant (according to the *p*-value) terms in the Yin and Yang gene lists, respectively.

### 2.2. Curated Data Sets

To train sample-specific models, data sets from multiple platforms were curated and are summarized in Table 1. The sequence-based predicted TF-binding profile matrix between 795 TFs and 2492 distinct genes were downloaded from TRRUST v2.0 [16]. We also curated ChIP-seq TF-binding site data for 125 TFs from ReMap v1.2 [17]. Among the 125 TFs, 95 were derived from luminal A cell lines (with the majority (78) being MCF-7), 6 were from luminal B cell lines, 8 were from Her2-enriched cell lines, and 16 were from TN cell lines. The miRNA-binding profile matrix between 15,168 target genes and 600 miRNAs (see details in Materials and Methods section) was obtained from starBase v3.0 [18]. Based on the data from different platforms, we kept the common samples, Yin and Yang genes, and miRNAs for which data were available across different platforms. In the end, there were 391 common tumor samples with 3008 Yin/Yang genes, 795 TFs and 362 miRNAs (Table 1). For the 391 breast tumors, the expression, CNV and DM of the 3008 genes, and the expression of the 362 miRNAs were available. The interaction data between the 795 TFs or the 362 miRNAs and the 3008 genes were also obtained. Among the 391 tumors, 239 were Luminal A, 74 were Luminal B, 21 were Her2-enriched, and 57 were TN.

### 2.3. Construction of The Tumor-Specific Lasso Regression Models 

The lasso regularized linear regression approach was applied to the 391 breast tumor profiles, and a model was trained for each tumor independently. In the end, *W* = (*W_CN_*, *W_DM_*, *W_miR_*, *W_TF_*) was the vector of regression coefficients for each tumor. Combing the 391 tumor-specific models, the following matrices were obtained for further analyses: {*w*}***_N_*_×*M*_**, where *N* is the 391 tumors/models and *M* is the 1159 features (CNV, DM, 795 TFs and 362 miRNAs).

**Figure 3:**
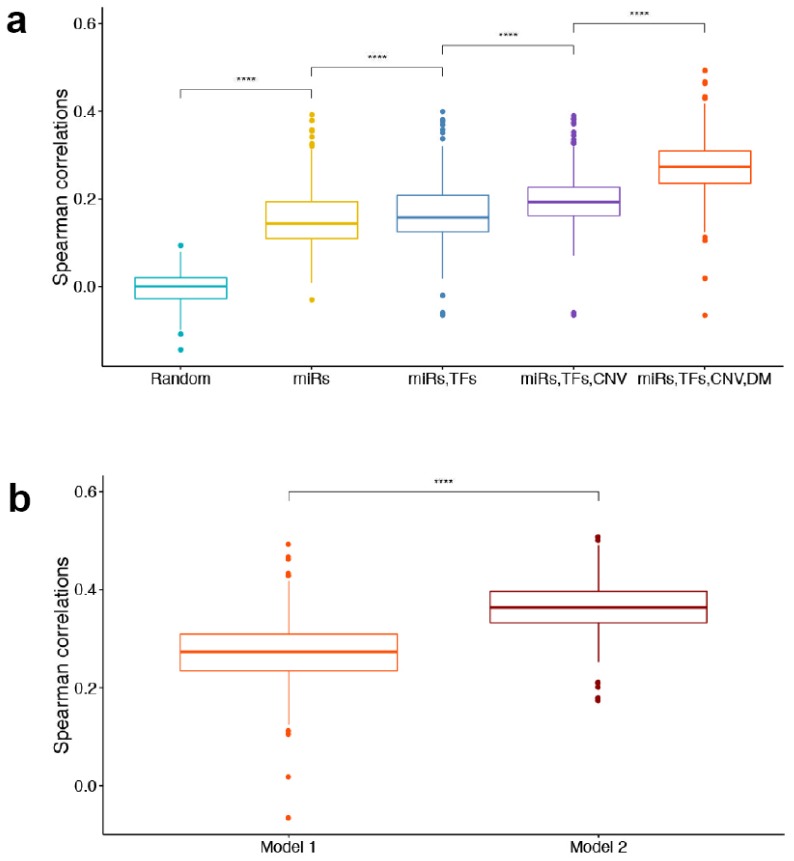
Model comparison. (**a**) The boxplot shows Spearman correlations between predicted and actual gene expression changes for all samples in models with different regulators. Using only miRNA-binding sites as features was significantly better than random; adding TF-binding sites significantly improved CV performance over using only miRNAs-binding sites; adding CNV significantly improved CV performance over using only TF- and miRNA-binding sites, while the model using TF/miRNA-binding sites, CNV and DM outperformed the others. (**b**) Boxplot shows Spearman correlations for all samples in Model 1 and Model 2. Model 1 was trained using CNV, DM, miRNA-binding sites and TF-binding sites from TRRUST, while Model 2 was trained using CNV, DM, miRNA-binding sites and TF-binding sites from ReMap. ^****^ *p*-value < 2.2 × 10^−16^ by Wilcoxon signed rank test.

To justify our model formalism, we compared models with different numbers of features in terms of their abilities to explain expression changes of the Yin and Yang genes in BC. Figure 3a illustrates the 10-fold cross validation (CV) results as a distribution of the correlation calculated across all 391 samples for each model. Unsurprisingly, the model with all features (red, right most in Figure 3a) performed the best and significantly better (*p*-value < 2.2 × 10^−16^, Wilcoxon signed rank test) than all the other alternatives, achieving a mean Spearman correlation of 0.273. Comparing the best model with other models (where some regulatory factors were excluded), it is remarkable to observe that a significant improvement in CV performance (*p*-value < 2.2 × 10^−16^, Wilcoxon signed rank test) is attributable to the addition of DNA methylation.

We further compared two alternative models using CNV, DM and miRNA-binding site data, but with different TF-binding site data from TRRUST (the model used in this study) and ReMap, respectively. We then compared the two models in terms of the Spearman correlation between the predicted and observed gene expression changes via 10-fold CV. We found that the second model using ReMap performed significantly better than the first model (*p*-value < 2.2 × 10^−16^, Wilcoxon signed rank test) (Figure 3b). Therefore, TF-target interaction data derived from the ChIP-seq method conferred significantly higher explanatory power than the binding motif-based TRRUST database for the Yin and Yang gene expression changes in BC. This is not surprising, because ReMap contains experimentally determined TF binding sites whereas TRRUST predicts potential TF binding sites. Nevertheless, we decided to use the TRRUST model because of the limited ChIP-seq TF-binding site data that were available. In addition, the majority of the ChIP seq data in ReMap were derived from MCF-7 and other luminal cell lines. Therefore, these data do not have representative cell lines from all of the BC types. Such overrepresentation of one BC type could bias any potential predictive capabilities of our model.

### 2.4. Identification of Key Regulators in Subtypes

To determine the most predominant TF/miRNA regulators that regulate Yin and Yang gene expression in different BC subtypes, we performed a feature selection procedure. With a threshold corresponding to an FDR of 0.05 relative to regression models trained on randomized data, we identified 25, 20, 15 and 24 key regulators for the luminal A, luminal B, Her2-enriched and TN subtypes, respectively. Table 2 summarizes the key shared and subtype-specific regulators for the four subtypes identified by our analysis. No significant regulators were found to be unique to the Her2-enriched subtype alone. Regulators that are shared among all four subtypes are considered to be common regulators, including 2 TFs (E2F1 and CITED2) and 7 miRNAs. E2F1, a member of the E2F transcription factors, plays a crucial role in the control of cell cycle and action of tumor suppressor proteins [19]. E2F1 was significantly upregulated in all four subtypes compared to normal samples in the TCGA data set (*p*-value < 0.05, *t*-test). CITED2 is a part of the CBP/p300 transcription complex and is known to increase *PPARA* transcription [20] and enhance ER mediated transcription [21]. It is known to be a potent prognostic predictor associated with proliferation, migration and chemo-resistance in breast carcinoma [22] and was significantly downregulated in BC compared to normal samples in the TCGA data set (*p*-value < 0.05, *t*-test).

Among the 7 common miRNAs, 3 miRNAs (hsa-miR-340-5p, hsa-miR-32-5p and hsa-miR-3150b-3p) were upregulated (*p*-value < 0.05, *t*-test), while 3 miRNAs (hsa-miR-374b-5p, hsa-miR-664b-3p and hsa-miR-296-5p) were downregulated (*p*-value < 0.05, *t*-test) in each of the four subtypes compared to normal samples. Only hsa-miR-361-3p showed no significant difference between each subtype and normal samples. Targets of hsa-miR-340-5p include oncogenes such as *KRAS* and *MET* [23]. hsa-miR-32-5p was previously reported to modulate vascular smooth muscle cell (VSMC) calcification progression by downregulating PTEN, and thus activating PI3K signaling and increasing RUNX2 expression and phosphorylation in mice [24]. High levels of hsa-miR-374b-5p have been observed to correlate with favorable outcomes in TNBC [25]. hsa-miR-296-5p was shown to play a tumor-suppressive role by directly targeting Pin1 in prostate cancer [26].

### 2.5. Identification of Key Regulators in TN Subtype

**Figure 4:**
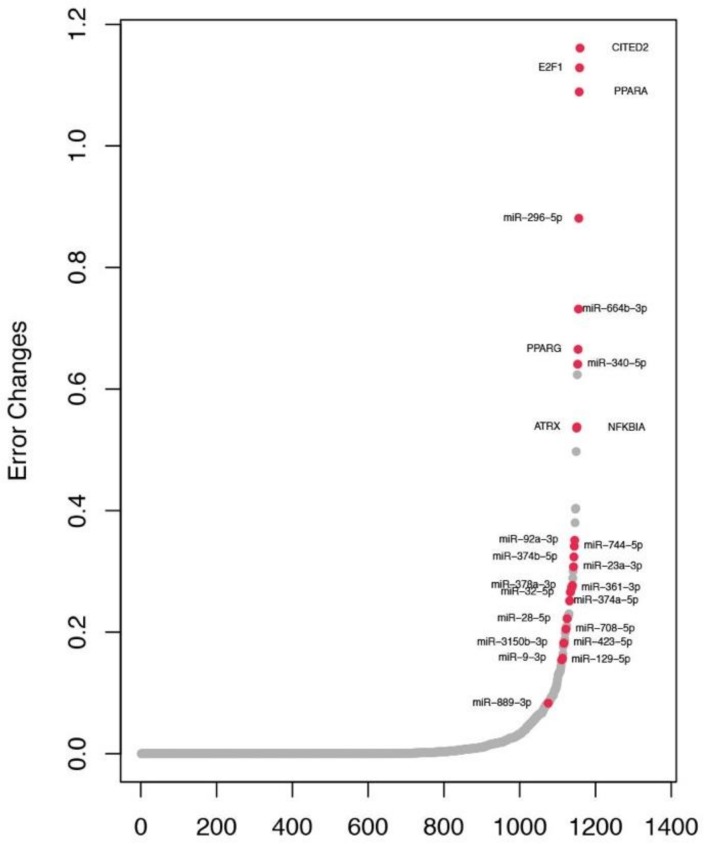
Error changes caused by regulators of the TN subtype. The x-axis indicates the miRNA/TF regulators, and the y-axis indicates increase in squared error across samples of the TN subtype after excluding the regulator from regression models. All regulators are ranked based on increase in squared error, and 24 key regulators (red dots) for the TN subtype were identified with FDR < 0.05.

Since there are no effective targeted drug therapies available for the TN subtype, it is necessary to investigate the key regulators in this subtype more closely. A total of 24 predominant regulators, consisting of 6 TFs and 18 miRNAs, satisfied a 5% FDR cutoff for the TN subtype. Figure 4 shows the results of the feature selection analysis for the TN subtype, with miRNAs and TFs sorted by the increase in total square loss incurred by their individual removal from the model. Most regulators were not chosen in any of the TN regression models and therefore led to zero change in loss, while only the 24 key regulators passed the FDR cutoff (red dots in Figure 4). To examine the biological functions of these TN regulators, we performed a functional enrichment analysis using their associated Yin and Yang targets. Based on enrichments for canonical pathways from the Molecular signatures database (MSigDB) [27], we discovered several interesting functions related to the TN regulators (FDR < 0.05, Table 3). For instance, 11 of the 24 selected regulators are engaged in pathways in cancer (KEGG_PATHWAYS_IN_CANCER) whereas 7 of the 24 regulators are involved in the cell-matrix adhesion pathway (KEGG_FOCAL_ADHESION) (see Table 3). Additionally, has-miR-340-5p and E2F1 are part of the RB1 pathway (PID_RB_1PATHWAY) and P53 pathway (PID_P53_DOWNSTREAM_PATHWAY).

In our previous study, we identified the top Yin (upregulated) FOXM1 and the top Yang (downregulated) PPARA pathways [28]. Remarkably, in this study, we found that six TN regulators (PPARG, PPARA, hsa-miR-9-3p, hsa-miR-664b-3p, hsa-miR-340-5p and hsa-miR-129-5p) regulate the PPARA pathway (BIOCARTA_PPARA_PATHWAY), while two TN regulators (hsa-miR-340-5p and E2F1) regulate the FOXM1 pathway (PID_FOXM1_PATHWAY). In addition, targets of PPARG, PPARA and hsa-miR-340-5p are enriched in the PPAR pathway (KEGG_PPAR_SIGNALING_PATHWAY), which has similar mechanisms to the PPARA pathway. This further supports the importance of the PPARA and FOXM1 pathways and their regulators in TNBC.

### 2.6. Regulatory Network Involving The TN Regulators 

**Figure 5:**
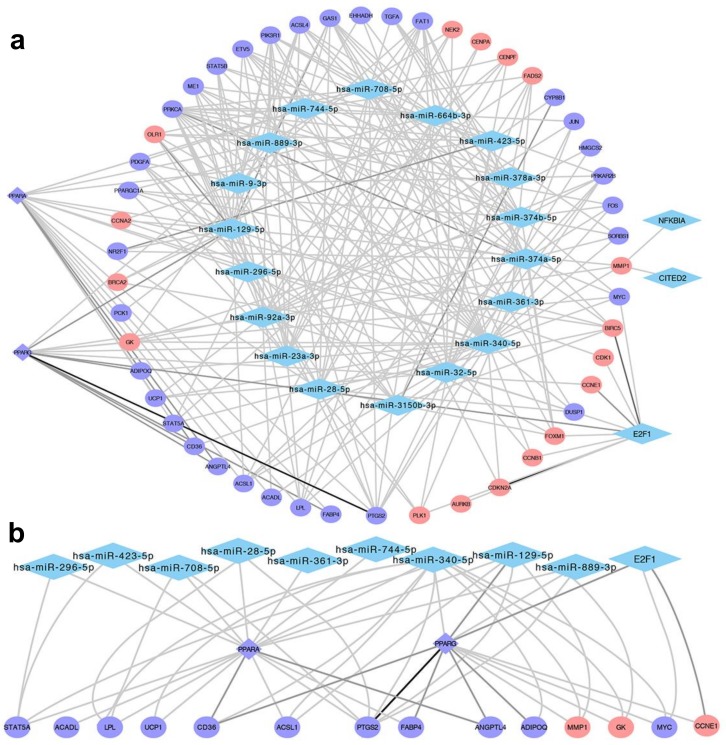
TF-target and miRNA-target regulatory networks. (**a**) The network comprises 23 selected regulators (21 blue diamonds and 2 red diamonds) with nonzero predicted interactions with PPAR/FOXM1 pathway-related genes (circles) obtained from MSigDB. The red and purple circles indicate Yin (upregulated) and Yang (downregulated) genes, respectively. The color of edges ranging from grey to black corresponds to an order of increasing number of hits. (**b**) A subnetwork contains PPARA and PPARG, their targets, and regulators that regulate them. A 2-layer hierarchical structure forms with 10 upstream regulators, including 9 miRNAs and E2F1 on the top layer, and two downstream regulators PPARA and PPARG arranged at the bottom layer.

To visualize the regulatory relationships between the TN regulators and their target Yin and Yang genes involved in the PPARA, PPAR and FOXM1 pathways, the interactions between 23 TN regulators and 51 Yin/Yang genes were imported into Cytoscape v3.5.1 [29] to construct a network. In Figure 5a, the resulting regulatory network is densely connected, implying that the TN regulators we identified shared many common Yin and Yang target genes involved in the PPARA, PPAR and FOXM1 pathways. We examined the hierarchical organization of the select subnetwork of PPARA and PPARG regulators. A two-layer network architecture formed with PPARA and PPARG, their targets, and the regulators that regulate their expression (Figure 5b). Specifically, 9 miRNAs and E2F1 form the top layer (master regulators), and PPARA and PPARG from the bottom layer (regulators that are regulated by the master regulators above it and regulate other Yin and Yang targets below it). Remarkably, the fact that 9 miRNAs regulate PPARA and PPARG and a large cohort of the PPARA, PPAR and FOXM1 pathway-related Yin and Yang genes either directly via base-pairing or indirectly via PPARA and PPARG provides further support of the important role of these 9 miRNAs as master regulators of the PPARA, PPAR and FOXM1 pathways in TNBC.

### 2.7. Clinical Relevance of The TN Regulators 

**Figure 6:**
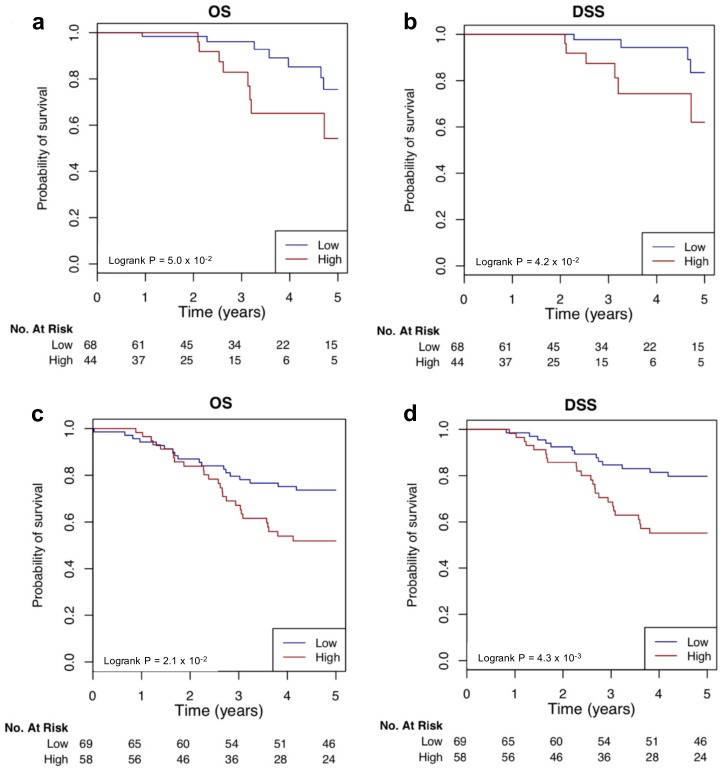
Survival analysis of the 31 gene YMR signature. TN patients were divided into high-risk (“High,” red line) and low-risk (“Low,” blue line) groups according their YMR signature scores. Survival fractions as a function of survival time (years) were then plotted for the two groups and the significant separation of the two curves were assessed by log-rank test. The YMR signature was tested using TNBC samples of TCGA and METABRIC datasets separately by the R package survcomp. The YMR signature can significantly stratify the 112 TCGA TNBC samples into high- and low-risk groups by both overall survival (OS) (**a**) and disease specific survival (DSS) (**b**). The YMR signature can also significantly stratify the 127 METABRIC TNBC samples into high- and low-risk groups by both OS (**c**) and DSS (**d**).

To determine if Yin and Yang genes selected from the PPARA, PPAR and FOXM1 pathways were clinically relevant, we tested if they can be used to develop multigene signatures for TNBC prognosis. Yin (17) and Yang (34) genes from the pathways were then used to calculate the Yin Yang gene expression mean ratio (YMR) signature, which was developed previously [14]. The 51 gene YMR signature stratified the 112 TCGA TN samples into high- or low-risk groups with significant accuracy evaluated by both five-year overall survival (OS) (*p*-value = 5.0 × 10^−2^, log-rank test) (Figure 6a) and disease-specific survival (DSS) (*p*-value = 4.2 × 10^−2^, log-rank test) (Figure 6b). We further tested the YMR using the Breast Cancer International Consortium (METABRIC) TNBC dataset [30]. As shown in Figure 6c,d, the YMR signature significantly stratified the 127 TN patients into high- and low- risk OS (*p*-value = 2.1 × 10^−2^, log-rank test) and DSS groups (*p*-value = 4.3 × 10^−3^, log-rank test).

**Figure 7:**
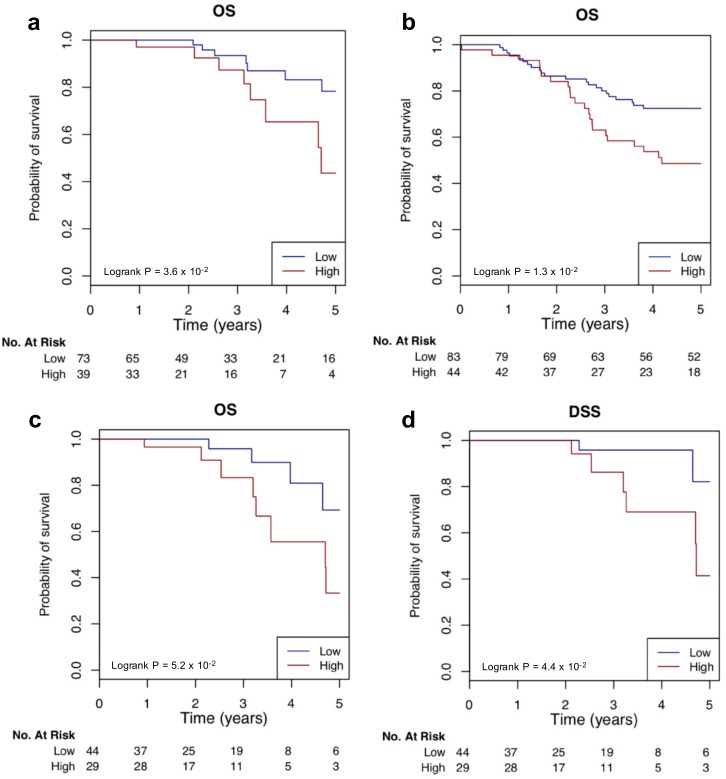
Kaplan–Meier survival analysis of the combined risk score. TN patients were divided into high-risk (“High,” red line) and low-risk (“Low,” blue line) groups according the 6 TF or 24 regulator combined risk scores. Survival fractions as a function of survival time (years) were then plotted for the two groups, and the significant separation of the two curves was assessed by log-rank test using the R package survcomp. The 6 TF combined risk score significantly stratified the TNBC samples from TCGA (**a**) and METABRIC (**b**) into high- and low-risk OS groups, respectively. The 24-regulator combined risk score significantly stratified the 112 TCGA TNBC samples into high- and low-risk groups in terms of OS (**c**) and DSS (**d**).

We next examined the implication of the expression of the above identified 24 regulators. We first evaluated the prognostic value of each regulator. Eight regulators showed a significant stratification in survival outcomes for TCGA TNBC patients (Supplementary file: Figure S1), and 11 regulators could significantly stratify TCGA BC patients (Supplementary file: Figure S2-1 and S2-2). 4 regulators (ATRX, NFKB1A, hsa-miR-23a-3p and hsa-miR-708-5p) could be a prognostic marker for both BC and TNBC patients. Combined risk score (CRS) models were then built using different TN regulators. The CRS computed from the 6 TFs could assign TN patients into high- and low-risk groups. The two groups showed a significant difference in OS time in both TCGA (*p*-value = 3.6 × 10^−2^, log-rank test) (Figure 7a) and METABRIC (*p*-value = 1.3 × 10^−2^, log-rank test) (Figure 7b) data sets. In addition, the CRS with all 24 regulators showed high-risk and low-risk group stratification significantly in terms of both OS (*p*-value = 5.2 × 10^−2^, log-rank test) (Figure 7c) and DSS (*p*-value = 4.4 × 10^−2^, log-rank test) (Figure 7d). However, we did not further validate the 24 regulator CRS signature due to the fact that we could not obtain gene and miRNA expression data for the 24 regulators for TN cases from other data sets.

## 3. Discussion

In this study, instead of building gene-specific models as done in most other studies [31], we built a sample-specific model to identify the key common and BC subtype-specific regulators for Yin and Yang genes with opposing effects. Remarkably, most of the identified regulators had been previously identified as being involved in breast carcinogenesis in the published literature. Among the TN regulators, PPARA and PPARG are two members of peroxisome proliferator-activated receptors (PPARs) that belong to the nuclear hormone receptor (HR) superfamily, which includes ERs and PRs [32]. PPARs affect the expression of target genes involved in cell proliferation, cell differentiation, and in immune and inflammation responses [33]. A genetic variant of *PPARA* has been linked to BC risk [34]. NFKBIA inhibits the oncogene nuclear factor-kappaB (NFkB). NFkB is constitutively activated in ER−negative and TN breast tumors, mediates adaptive resistance to ionizing radiation and chemotherapy, and is required for epithelial–mesenchymal transition (EMT) associated with metastatic progression of BC. *NFKBIA* deletions, leading to a haploinsufficient effect on NFKBIA expression and thus the transactivation of several NFkB target genes with important roles in TN breast carcinogenesis, are significantly associated with TN patients [35]. hsa-miR-423-5p has been observed to be upregulated in drug-resistant BC cells from the parental MDA-MB-231 cell line [36]. Downregulation of hsa-miR-92a-3p is associated with aggressive BC features and increased tumor macrophage infiltration [37]. hsa-miR-129-5p was reportedly downregulated in BC cells, in part because of promoter H3K27 methylation and also because it regulates EMT and multi-drug resistance [38]. One of the hsa-miR-129-5p target genes is *CBX4*, which is up-regulated in BC tissues and thus promotes cell proliferation [39]. The TN-specific regulator hsa-miR-9-3p has been observed as a novel tumor suppressor in gastric cancer [40]. In vitro, hsa-miR-9-3p has also been identified as a tumor-suppressor miRNA targeting β1 integrin in claudin-low TNBC cells, resulting in the sensitization of MDA-MB-231 cells to MEK inhibition [41]. There are seven TN miRNA regulators (hsa-miR-378a-5p, hsa-miR-708-5p, hsa-miR-889-3p, hsa-miR-3150b-3p, hsa-miR-374a-5p, hsa-miR-23a-3p and hsa-miR-28-5p), the targets of which are not enriched in canonical pathways. Many of them are also engaged in breast tumorigenesis. hsa-miR-378a-5p is believed to be a switch regulating the Warburg effect in BC [42]. Studies in BC have shown that hsa-miR-708-5p is a tumor suppressor through repression of invasion, proliferation, and potentially immune modulation [43]. hsa-miR-374a-5p could constitutively activate Wnt/β-catenin signaling to promote BC metastasis, and thus may serve as an anti-metastasis therapeutic target in BC [44]. hsa-miR-23a-3p was shown to be up-regulated in a variety of human cancers including BC and to exert an oncogenic function in human tumorigenesis [45].

When comparing models with different features, a significant improvement in CV performance was observed when DNA methylation was added to the models. DNA methylation for early detection and prognosis of breast cancer has been a recent topic of study. Fackler et al. found that promoter methylation of 4 genes (*RASSF1A*, *CCND2*, *TWIST* and *HIN1*) was more frequently detected in breast tumors than in normal tissue [46]. In another study, 10 hyper-methylated genes (*APC*, *BIN1*, *BMP6*, *BRCA1*, *CST6*, *ESR-b*, *GSTP1*, *CDKN2A*, *P21* and *TIMP3*) were identified to distinguish between BC and normal tissues [47]. In our study, we identified *CCND2*, *TWIST*, *BIN1*, *BMP6* and *TIMP3* as Yang genes, underscoring the fact that DNA methylation is an important factor for explaining the Yin and Yang gene expression changes in BC.

It is noteworthy that many of the TN regulators were found to regulate the FOXM1 and PPARA pathways, which were identified as the top Yin (upregulated) and the top Yang (downregulated) pathways, respectively, in TNBC in our pervious study [28]. Stimulation of the PPARA pathway increases the volume and number for peroxisomes, which are responsible for, among other things, lipid metabolism. This pathway can also induce tumor cell apoptosis and includes 58 genes, such as the tumor suppressors *RB1* and *PIK3R1*, oncogenes *MYC* and *JUN*, as well as TFs *CITED2* and *PPARA*. Two TNBC TFs, PPARA and PPARG, were found to regulate the PPARA pathway. PPARA and PPARG ligands, known as peroxisome proliferators, have been shown to induce cell cycle arrest at the G1 phase of the cell cycle to prompt the differentiation of liposarcoma, colon, prostate and BC cells, conferring a less malignant phenotype to the cells [28]. PPARA and PPARG are also involved in the regulation of cholesterol metabolism [48], which was shown to regulate cell adhesion in breast cancer cell lines [49].

The FOXM1 pathway involves both the cell cycle and DNA damage repair ultimately promoting tumor cell proliferation. Forty genes are engaged in this pathway, including tumor suppressors (such as *BRCA2*, *CDKN2A*, *CHEK2* and *RB1*), oncogene MYC, cyclins (*CCNA2*, *CCNB1*, *CCNB2*, *CCND1* and *CCNE1*), cyclin-dependent kinases (*CDK1*, *CDK2* and *CDK4*), *ESR1*, *FOXM1* as well as *NEK2*. NEK2, a cell cycle dependent serine-threonine kinase, was also identified as a Yin gene in this study, and is regulated by 4 of the 24 TN regulators. *NEK2* has been shown to be upregulated in cancers such as lymphoma, cholangiocarcinoma, breast, prostate and cervical [50]. NEK2 functions in the regulation of mitotic spindle formation, chromosome segregation, cell division, carcinogenesis and the tumorigenic growth of breast cancer [51,52]. FOXM1 is one of the most important oncogenic transcription factors overexpressed in various human cancers [53]. It regulates all hallmarks of cancer, including proliferation, mitosis, EMT, invasion and metastasis [54]. FOXM1 has been found to be overexpressed in 85% of TNBCs [53], and has been identified as the key transcriptional driver in the differentially expressed gene signature of TNBCs [55]. FOXM1 could promote TNBC proliferation, invasion and tumorigenesis by directly binding to and transcriptionally regulating expression of eukaryotic elongation factor 2 kinase (eEF2K) [53]. FOXM1 also plays a role in autophagy by transcriptionally regulating the *Beclin-1* and *LC3* genes in TNBCs [56]. Therefore, inhibition of FOXM1 transcription factor function is a potential strategy for overcoming TNBC progression.

A limitation of this study is the use of the motif-based TF binding data. Studies have shown that the ChIP-seq TF binding data conferred significantly higher explanatory power than the motif-based TF binding data for mRNA expression levels [31]. We observed the same phenomenon, finding that experimentally derived ChIP seq data from ReMap resulted in a significantly higher spearman correlation than the motif based TRRUST (Figure 3b). Unfortunately, the ChIP-seq data from ReMap were available for only a subset of the TFs in a limited number of BC cell lines that did not have representatives from each BC subtype. In fact, the majority were from MCF-7 cell lines. Therefore, a reasonable alternative for us was to use the motif-based TF-target interaction data, since it is not representative of any particular BC cell subtype.

## 4. Materials and Methods

### 4.1. TCGA Data Collection

The TCGA multi-omics Level 3 processed data including mRNA/miRNA expression profiles, DM and CNV data, were retrieved for breast tumor samples from the UCSC Xena browser (http://xena.ucsc.edu/). Only mRNA/miRNA expression data were downloaded for normal samples. Expression data for mRNA were transformed in RNA-seq by Expectation-Maximization (RSEM) values from the Illumina HiSeq 2000 RNA Sequencing platform were transformed to log2(RSEM+1) to estimate the gene-level transcription. We included miRNA mature strand expression data by RNA-seq (IlluminaHiseq 2000). For each sample, all isoform expression in reads per million miRNAs mapped (RPM) values for the same miRNA mature strand were added together and the log2(total_RPM+1) transformation was applied to estimate the miRNA expression level. The miRNA IDs from miRBase Release 22.1 were used [57]. The mRNA and miRNAs with missing values in more than 50% of the tumor and normal samples were removed. For DM data, we used the Illumina methylation450 data and beta values as the methylation scores. When multiple scores were available for the same gene, we took the average. For CNV data, we used the whole genome microarray data, which were estimated at the gene level using the GISTIC2 method. In addition, clinical data for BC patients were downloaded from the Firehose TCGA data portal (http://firebrowse.org/). Patients with information on their immunohistochemistry (IHC) status of three receptors (ER, PR, HER2) available were categorized into four molecular subtypes: (1) luminal A (ER+ and/or PR+ and HER2−); (2) luminal B (ER+ and/or PR+ and HER2+); (3) Her2-enriched (ER− and PR− and HER2+); (4) triple-negative (ER− and PR− and HER2−).

### 4.2. Target Prediction for miRNAs and TFs

To get the highest number of publicly available miRNA-target pairs into the regression models, we used the StarBase v3.0 database, which contains the amalgamation of miRNA–mRNA interactions predicted by seven algorithms (PITA, RNA22, miRmap, microT, miRanda, PicTar, TargetScan) [18]. In our study, among the miRNA–target gene pairs, those predicted by at least one algorithm were selected. Binding site predictions for human sequence-specific TFs based on motif hits were used and downloaded from TRRUST v2.0 [16].

We also curated ChIP-seq-derived TF binding data from ReMap v1.2 [17]. ReMap contains a catalog of regulatory regions by compiling the genomic localization of 485 different transcriptional regulators (TRs) across 346 different human cell lines and tissue types. This database is based on the latest 1066 Encyclopedia of DNA Elements (ENCODE) ChIP-seq datasets [58], as well as 1763 non-ENCODE datasets selected from Gene Expression Omnibus [59] and ArrayExpress [60]. In total, ChIP-seq data were obtained for 125 TFs across 13 diverse untreated BC cell lines. We defined the promoter region for each gene as 2000 bp upstream to 200 bp downstream from the transcription start site (TSS), based on *Homo sapiens* (GRCh38/hg38) annotation. We then annotated the peaks within promoters for each TF using the R package ChIPpeakAnno [61], and averaged the TF binding scores for multiple peaks to represent a single binding site per TF–gene pair.

### 4.3. Identification of Yin and Yang Genes

DEGs were determined using the R package limma [62] with the gene expression values for the tumor (*n* = 1104) and normal breast tissue (*n* = 114) samples from TCGA. DEGs with FDR < 0.05 and an absolute value of logFC not less than 1 were considered to be Yin and Yang genes and were selected for further analysis.

### 4.4. Tumor-Specific Lasso Regression Models

We trained linear regression models separately for each tumor sample to explain log gene expression fold changes (tumor versus normal tissue), using CNV, TF binding site counts in the gene’s promoter, and miRNA binding site counts in the gene’s 3′UTR as covariates in the models. Tumor-specific miRNA expression fold changes (tumor versus normal tissue) were used to restrict the miRNAs that can be used as explanatory variables. Specifically, suppose there are *N* tumor samples, *G* Yin and Yang genes (*g*), *M* miRNAs and *T* TFs. For each sample *n*, we observed the log expression fold change in the tumor (relative normal tissue), CNV and DM value for gene *g,* as well as the log expression fold change in the tumor (compared to normal tissue) for all the *M* miRNAs (together provided by TCGA). The binding site count for TFs or miRNAs in the gene’s promoter or 3′UTR, however, were not available for each sample, but rather were estimated by sequence-derived methods using the TRRUST v2.0 [16] and starBase v3.0 [18], respectively. In this study, *N* = 391, *G* = 3008, *M* = 362 and *T* = 795.

To avoid overfitting in the presence of noisy expression data and a large number of explanatory variables, regularized regression via a lasso constraint [63] was used to identify a small number of TFs and miRNAs that best explain changes in gene expression on a sample-by-sample basis. The lasso constraint enforces sparsity in the learned parameters, with the result that most of the regression coefficients are zero. This reduces the number of features included in the model, leading to better prediction accuracy and more interpretable results. The sample-by-sample approach trains a regression model for each tumor sample independently, and does not use information about the tumor’s assignment to previously defined transcriptomic subtypes. The regularization parameter λ controls the degree of sparsity in the trained model was determined by 10-fold CV.

The regression coefficient of each regulator (TF, miRNA) establishes the importance of the corresponding regulatory element for the prediction of gene expression changes, while the sign of the coefficient can be interpreted as the predicted direction of regulation. This can be formulated as a regression model with equation (1), where *y_g_* is the log expression fold change in the tumor (relative normal tissue) for gene *g*; *C_g_* and *D_g_* are the gene’s CNV and DM values, respectively; *N_g,TF_* and *N_g, miR_* are the number of binding sites for TF or miRNA in the gene’s promoter or 3′UTR, respectively; and *W* = (*W_CN_*, *W_DM_*, *W_miR_*, *W_TF_*) is the model vector of regression coefficients.

(1) $$y_{g}\;={\;W}_{\mathrm{CN}}C_{g}\;+{\;W}_{\mathrm{DM}}D_{g}\;+\;\underset{\mathrm{TF}\;\in \left\{1,\;\ldots ,\;T\right\}}{\sum }W_{\mathrm{TF}}N_{g,\mathrm{TF}}\;+\;\underset{\mathrm{miR}\;\in \;\left\{1,\;\ldots ,\;M\right\}}{\sum }W_{\mathrm{miR}}N_{g,\mathrm{miR}}\;$$

We further compared the performance of models trained with different numbers of features. The sample-specific models were first built with only miRNAs-binding sites as features. The other features, TFs-binding sites, CNV and DM, were added to the models one by one. For each modeling method and each sample, the Spearman correlation was computed using 10-fold CV on held-out genes. Specifically, for each tumor, we performed a 10-fold CV by training a model with given features on 90% of the genes and testing its predictions on the remaining 10% of genes. After each CV run, we obtained the Spearman rank correlation between the predicted and the observed Yin and Yang gene expression changes for the model in the tumor. In this way, the mean CV Spearman rank correlation was obtained for each model in each tumor. By contrast, we randomized the output gene expression changes, then trained sample-specific regression models.

### 4.5. Feature Selection to Identify the Key Regulators

Similar to the definition in reference [12], a feature selection procedure was performed using a scoring technique. Specifically, for a given subtype *S*, the regression coefficient of each single regulator *r* (TF or miRNA) was set to zero for all samples belonging to this subtype *S,* and the change in total squared loss *L* over all genes was computed in these samples as *score(r, S)*. The formula is as follows:

(2) $$score\left(r,\;S\right)\;=\;\underset{g\;\in \left\{1,\;\ldots ,\;G\right\}}{\sum }score\left(r,S,\;g\right)=\underset{g\;\in \;\left\{1,\;\ldots ,\;G\right\}}{\sum }\;\underset{k\;\in \;S}{\sum }\left\{L\left(y_{g,k},W_{k}^{r\to 0}\cdot X_{g,k}\right)-L\left(y_{g,k},W_{k}\cdot X_{g,k}\right)\right\}\;\;$$

where *k* indexes the tumor samples and *g* indexes the Yin and Yang genes, $y_{g,\;k}$ is the expression change of gene g in sample *k*, $X_{g,\;k}$ is the vector of TF and miRNA binding site counts for gene *g* and the CNV and DM of gene *g* in sample *k*, and *S* ranges over the set of the four subtypes. *L* is squared loss and $W_{k}^{r\to 0}$ denotes the model vector obtained from $W_{k}$ by setting the coefficient $W_{k}^{r}$ to 0. This score measures the degree of influence of the regulator in predicting the changes in gene expression. The high-scoring regulators, the removal of which caused large increases in loss over the samples in each BC subtype, were considered key regulators.

To further assess the statistical significance of the feature scores, we derived the randomized data by permuting the motif hit over the genes independently for each TF/miRNA. We carried out the tumor-specific lasso training procedure on randomized data 5000 times, and then computed the resulting random score distribution for each regulator and each subtype. The empirical *p*-value for each regulator and subtype was calculated based on these distributions and adjusted for multiple testing over the TF/miRNA regulators using the Benjamini–Hochberg (BH) method to produce FDR. Subtype-specific key regulators were finally selected with FDR < 0.05.

### 4.6. Network Construction

To construct the network, the TF/miRNA–gene pairs involving nonzero binding hits between the 24 TN regulators and all the identified Yin and Yang genes were first compiled. We then extracted the gene sets of the three pathways (BIOCARTA_PPARA_PATHWAY, KEGG_PPAR_SIGNALING_PATHWAY and PID_FOXM1_PATHWAY) from MSigDB [27] and filtered the regulator–gene pairs by gene sets of the three pathways. A matrix (262 rows by 3 columns), containing 262 interactions between 23 TN regulators and 51 Yin/Yang genes, was thus generated. In the matrix, the first column is the selected TN regulators, used as the source node for the network; the second column is the corresponding targets, which are members of at least one of the three pathways and used as the target nodes for the network; the third column is the number of hits between the regulator–target pairs and used as the interaction for the network. Finally, the matrix was imported into Cytoscape v3.5.1 [29] to generate the network.

### 4.7. Gene Annotation

Gene ontology (GO) annotation was performed on the selected Yin and Yang genes using the R package clusterProfiler [15], with respect to the GO terms in Biological process (BP) (GO-BP) as the reference gene sets. The hypergeometric method was used to find the over-represented GO-BP terms in the identified Yin and Yang genes. The maximum gene set size was 500, while the minimum gene set size was 10. The resulting *p*-values were adjusted for multiple testing with the BH method. GO-BP terms with an adjusted *p*-value smaller than 0.05 were considered to be significantly over-represented in the Yin or Yang genes.

We also performed a functional enrichment analysis using targets of the selected TN regulators. Specifically, we extracted the predicted targets with nonzero binding sites provided by TRRUST v2.0 (starBase v3.0) for each of the selected TFs (miRNAs). For each regulator, the target list was intersected with the identified Yin and Yang genes, and only targets that were also Yin/Yang genes were kept for the regulator. We then downloaded canonical pathways from MSigDB (c2.cp.v6.2.entrez.gmt) [27]. For each target list, we assessed their enrichment for each canonical pathway with a hypergeometric test using clusterProfiler [15]. The maximum gene set size was 500, while the minimum gene set size was 2. The multiple test *p*-value was adjusted by the BH method to produce FDR, and we identified pathways related to the selected regulators with enrichment where FDR < 0.01.

### 4.8. Potential Clinical Outcome Association Analysis

Datasets from the TCGA [55] and METABRIC [30] databases were used to analyze clinical outcome associations. For TCGA BC patients, curated clinical TCGA data from a study [64] were downloaded from UCSC Xena. The study highlighted four types of carefully curated survival endpoints, and recommended the use of the endpoints of overall survival (OS), disease-specific survival (DSS), progression-free interval (PFI) and disease-free interval (DFI) for each TCGA cancer type. For METABRIC, the discovery set was used. The normalized gene expression levels were obtained from the European Genome-Phenome Archive (https://ega-archive.org/dacs/EGAC00001000484), and the curated clinical data were downloaded from a previous study [30]. TN cases (127 out of 997 breast cancer cases) with survival data (OS and DSS) and normalized gene expression profiles were selected for the following survival analysis.

To examine whether each of the 24 TNBC regulators alone could be a prognostic marker for BC patients, we performed survival analysis for each regulator in the TNBC and BC cohorts from TCGA. The mean expression level of a given regulator was used as the cutoff to stratify patients into two groups. Kaplan–Meier (KM) survival analysis with different endpoints (OS, DSS, PFI, DFI) was performed, using the R package survcomp [65] to examine the survival differences between the two groups.

To build the YMR signature [14], 17 Yin and 34 Yang genes that were members of at least one of the three pathways (PPARA, PPAR and FOXM1) and were targets of at least one of the 24 TN regulators were selected. The YMR was built using the 112 TCGA TN samples. Specifically, the YMR signature score was computed for each case independently by using the ratio of the mean of the 17 Yin genes to the mean of the 34 Yang genes. The TN samples were then stratified into high-risk and low-risk groups using the mean of the YMR scores as the cutoff. Cases with a YMR score above the cutoff were assigned to the high-risk group, while cases below the cutoff were grouped into low-risk group. We performed KM survival analysis to examine the prognostic power of the YMR signature. We further evaluated the reproducibility of the 31 gene YMR signature by testing the statistical association of the YMR scores with the clinical outcome in the 127 TNBC samples of METABRIC discovery set.

Additionally, we built the CRS signature using the 6 TFs of the 24 regulators. For each TF, the association between its expression and the patient’s OS outcome was determined using the univariate Cox proportional hazards (Cox) model [66] in the 112 TCGA TN samples, and the coefficient was extracted from the Cox model. With the expression (*x_i_*) and the corresponding coefficient (*coef_i_*) of the 6 TFs, we calculated the CRS as sum (*x_i_* × *coef_i_*). TN patients with CRSs were binarized into high-risk and low-risk groups using the *xtile* function in the R package statar with a probability parameter set to 0.65. The survival differences between the high- and low- risk groups were assessed by KM curves. We further tested the prognostic power of the 6-TF CRS signature in METABRIC TNBC data. In the same way, the CRS was computed using all 24 regulators for TN samples in TCGA data set.

## 5. Conclusions

Using the lasso regularized linear regression approach, we identified the subtype-specific as well as common TFs/miRNAs in BC. Many of the regulators identified in TNBC were found to regulate the FOXM1, PPARA and PPAR pathways. Therefore, further exploring these pathways and their regulators potentially could provide novel drug targets that may yield new treatment options for TNBC patients. The Yin and Yang genes from the three pathways can also be used to build a multigene YMR signature to stratify TNBC further for prognosis. The resulting TNBC groups exhibit different clinical outcomes, which supports the utility of our approach.

## Figures and Tables

**Table 1:** Multi-dimensional data used to build the models.

Type	Platform/Category	Database	All Data	Common Data
Subtype annotation	IHC	TCGA	694 tumors: 424 LumA, 121 LumB, 37 Her2+, 112 TN	391 tumors: 239 LumA, 74 LumB, 21 Her2+, 57 TN
mRNA expression ^1^	Illumina Hiseq 2000	TCGA	17675 genes × 1104 tumors	3008 genes × 391 tumors
miRNA expression ^2^	Illumina Hiseq 2000	TCGA	600 miRNAs × 756 tumors	362 miRNAs × 391 tumors
Copy number variation (CNV)	Affymetrix SNP 6.0	TCGA	24776 genes × 1080 tumors	3008 genes × 391 tumors
DNA methylation (DM)	Illumina Infinium HumanMethylation450	TCGA	26586 genes × 790 tumors	3008 genes × 391 tumors
TF-target	Sequence-based	TRRUST v2.0	2492 genes × 795 TFs	3008 genes × 795 TFs
miRNA-target	Sequence-based	starBase v3.0	15168 targets × 618 miRNAs	3008 genes × 362 miRNAs

^1^ 114 normal samples were available for calculating Yin and Yang gene expression change; ^2^ 76 normal samples were available for calculating miRNA expression change

**Table 2:** Selected common and subtype-specific regulators.

Subtype	Number of Regulators	Regulator
Her2+, LumA, LumB, TN	9	E2F1, CITED2, hsa-miR-374b-5p, hsa-miR-32-5p, hsa-miR-3150b-3p, hsa-miR-361-3p, hsa-miR-340-5p, hsa-miR-664b-3p, hsa-miR-296-5p
Her2+, LumA, LumB	1	CEBPZ
LumA, LumB, TN	5	ATRX, PPARG, hsa-miR-374a-5p, hsa-miR-92a-3p, hsa-miR-744-5p
Her2+, LumA, TN	3	PPARA, hsa-miR-889-3p, hsa-miR-423-5p
LumA, LumB	1	hsa-miR-429
LumA, TN	5	NFKBIA, hsa-miR-23a-3p, hsa-miR-378a-3p, hsa-miR-129-5p, hsa-miR-28-5p
Her2+, LumB	1	hsa-miR-760
Her2+, TN	1	hsa-miR-708-5p
LumA	1	hsa-miR-181b-5p
LumB	3	hsa-miR-655-3p, hsa-miR-654-5p, hsa-miR-625-5p
TN	1	hsa-miR-9-3p

**Table 3:** Selected TN regulators.

Regulators	FDR	Expression ^1^	Targets ^2^	Enriched Pathways ^3^	Ratio ^4^	Enrichment FDR ^5^
E2F1	0.00	Up	48	REACTOME_CELL_CYCLE	19/241	4.43 × 10^–12^
				PID_FOXM1_PATHWAY	9/40	1.70 × 10^–11^
				KEGG_PATHWAYS_IN_CANCER	9/328	2.09 × 10^–5^
CITED2	0.00	Down	2	REACTOME_EXTRACELLULAR_MATRIX_ORGANIZATION	2/87	8.03 × 10^–4^
				REACTOME_DEGRADATION_OF_THE_EXTRACELLULAR_MATRIX	2/29	1.74 × 10^–4^
				NABA_ECM_REGULATORS	2/238	4.04 × 10^–3^
PPARA	0.00	Down	20	REACTOME_METABOLISM_OF_LIPIDS_AND_LIPOPROTEINS	11/478	8.87 × 10^–8^
				BIOCARTA_PPARA_PATHWAY	4/58	2.08 × 10^–4^
				KEGG_PATHWAYS_IN_CANCER	4/328	3.76 × 10^–2^
PPARG	0.02	Down	29	KEGG_PATHWAYS_IN_CANCER	9/328	5.73 × 10^–5^
				REACTOME_HORMONE_SENSITIVE_LIPASE_HSL_MEDIATED_TRIACYLGLYCEROL_HYDROLYSIS	3/13	5.48 × 10^–4^
				REACTOME_METABOLISM_OF_LIPIDS_AND_LIPOPROTEINS	8/478	3.77 × 10^–3^
				BIOCARTA_PPARA_PATHWAY	3/58	1.92 × 10^–2^
				REACTOME_DEGRADATION_OF_THE_EXTRACELLULAR_MATRIX	2/29	4.81 × 10^–2^
ATRX	0.05	Down	2	BIOCARTA_AHSP_PATHWAY	2/13	1.97 × 10^–6^
NFKBIA	0.05	Down	3	REACTOME_DEGRADATION_OF_THE_EXTRACELLULAR_MATRIX	3/29	9.65 × 10^–7^
				REACTOME_EXTRACELLULAR_MATRIX_ORGANIZATION	3/87	1.40 × 10^–5^
				KEGG_PATHWAYS_IN_CANCER	2/328	1.37 × 10^–2^
hsa-miR-374b-5p	0.00	Down	589	KEGG_PATHWAYS_IN_CANCER	28/328	4.84 × 10^–2^
hsa-miR-32-5p	0.00	Up	523	KEGG_FOCAL_ADHESION	19/201	4.45 × 10^–2^
				NABA_CORE_MATRISOME	22/275	4.74 × 10^–2^
hsa-miR-361-3p	0.00	-	488	KEGG_FOCAL_ADHESION	23/201	8.25 × 10^–5^
				KEGG_PATHWAYS_IN_CANCER	27/238	2.41 × 10^–3^
				PID_AVB3_INTEGRIN_PATHWAY	11/75	6.61 × 10^–3^
hsa-miR-340-5p	0.00	Up	927	KEGG_PATHWAYS_IN_CANCER	54/328	5.10 × 10^–9^
				PID_AVB3_INTEGRIN_PATHWAY	19/75	2.64 × 10^−5^
				KEGG_FOCAL_ADHESION	31/201	1.47 × 10^–4^
				BIOCARTA_PPARA_PATHWAY	15/58	1.47 × 10^–4^
				REACTOME_EXTRACELLULAR_MATRIX_ORGANIZATION	13/87	4.14 × 10^–2^
				PID_FOXM1_PATHWAY	8/40	4.41 × 10^–2^
hsa-miR-664b-3p	0.00	Down	431	KEGG_FOCAL_ADHESION	18/201	9.09 × 10^–3^
				BIOCARTA_PPARA_PATHWAY	9/58	9.09 × 10^–3^
				KEGG_CYTOKINE_CYTOKINE_RECEPTOR_INTERACTION	19/267	4.57 × 10^–2^
hsa-miR-296-5p	0.00	Down	353	KEGG_LEUKOCYTE_TRANSENDOTHELIAL_MIGRATION	12/118	6.14 × 10^–3^
hsa-miR-423-5p	0.00	-	590	KEGG_PATHWAYS_IN_CANCER	30/328	1.43 × 10^–3^
				PID_AVB3_INTEGRIN_PATHWAY	11/75	1.94 × 10^–2^
				REACTOME_GROWTH_HORMONE_RECEPTOR_SIGNALING	6/24	3.31 × 10^–2^
hsa-miR-92a-3p	0.00	Down	524	KEGG_FOCAL_ADHESION	19/201	4.47 × 10^–2^
hsa-miR-129-5p	0.02	Down	775	KEGG_PATHWAYS_IN_CANCER	37/328	1.90 × 10^–4^
				KEGG_FOCAL_ADHESION	26/201	5.83 × 10^–4^
				REACTOME_EXTRACELLULAR_MATRIX_ORGANIZATION	13/87	1.41 × 10^–2^
				PID_AVB3_INTEGRIN_PATHWAY	12/75	1.41 × 10^–2^
				BIOCARTA_PPARA_PATHWAY	10/58	1.92 × 10^–2^
hsa-miR-744-5p	0.03	-	221	KEGG_PATHWAYS_IN_CANCER	15/328	1.26 × 10^–2^
				KEGG_FOCAL_ADHESION	12/201	1.20 × 10^–2^
				REACTOME_CELL_SURFACE_INTERACTIONS_AT_THE_VASCULAR_WALL	7/91	2.57 × 10^–2^
hsa-miR-9-3p	0.04	-	236	KEGG_PATHWAYS_IN_CANCER	14/328	2.98 × 10^–2^
				BIOCARTA_PPARA_PATHWAY	6/58	2.98 × 10^–2^
				KEGG_MAPK_SIGNALING_PATHWAY	12/267	3.19 × 10^–2^

^1^ The miRNA/TF expression pattern in 112 TN patients when compared to the 113 adjacent normal breast tissue samples (TCGA data set was used); ^2^ The number of predicted targets provided by TRRUST v2.0 or starBase v3.0 overlapped with Yin and Yang genes; ^3^ The enriched canonical pathways from MSigDB in the target Yin and Yang genes of the corresponding regulator; ^4^ The number of the targets involved in a pathway and the total number of genes involved in the pathway; ^5^ The BH method adjusted hypergeometric test *p*-value for the corresponding enriched pathway.

## Supplementary Materials

The following are available online at https://www.mdpi.com/2072-6694/11/4/507/s1, Figure S1: Survival analysis of the TNBC regulators in TNBCs. Based on gene expression levels of a given regulator, TN patients from TCGA were divided into over-expressed (“high-exp”, red line) and down-expressed (“low-exp”, blue line) groups. Survival analysis with different endpoints (OS, DSS, PFI, DFI) was performed for each regulator. Only significant results were shown. 8 regulators alone could be a prognostic marker for TNBC patients: (a) ATRX, (b) NFKBIA, (c) hsa-miR-23a-3p, (d) hsa-miR-664b-3p, (e) hsa-miR-708-5p, and (f) hsa-miR-889-3p. OS, overall survival; DSS, disease-specific survival; PFI: progression-free interval; DFI, disease-free interval, Figure S2-1: Survival analysis of the TNBC regulators in BCs. Based on gene expression levels of a given regulator, BC patients from TCGA were divided into over-expressed (“high-exp”, red line) and down-expressed (“low-exp”, blue line) groups. Survival analysis with different endpoints (OS, DSS, PFI, DFI) was performed for each regulator. Only significant results were shown. 11 regulators alone could be a prognostic marker for BC patients (six were shown here): (a) ATRX, (b) NFKBIA, (c) PPARG, (d) CITED2, (e) hsa-miR-378a-3p, and (f) hsa-miR-32-5p. OS, overall survival; DSS, disease-specific survival; PFI: progression-free interval; DFI, disease-free interval, Figure S2-2: Survival analysis of the TNBC regulators in BCs. Based on gene expression levels of a given regulator, BC patients from TCGA were divided into over-expressed (“high-exp”, red line) and down-expressed (“low-exp”, blue line) groups. Survival analysis with different endpoints (OS, DSS, PFI, DFI) was performed for each regulator. Only significant results were shown. 11 regulators alone could be a prognostic marker for BC patients (five were shown here): (g) hsa-miR-3150b-3p, (h) hsa-miR-23a-3p, (i) hsa-miR-708-5p, (j) hsa-miR-129-5p, and (k) hsa-miR-92a-3p. OS, overall survival; DSS, disease-specific survival; PFI: progression-free interval; DFI, disease-free interval.
