# Supplementary material for: Integrative Analysis Reveals Subtype-Specific Regulatory Determinants in Triple Negative Breast Cancer
Source: Cancers (Basel). 2019 Apr 10;11(4):507. doi: 10.3390/cancers11040507 (PMC6521146; doi:10.3390/cancers11040507)
Supplement: Supplementary file 1 [file cancers-11-00507-s001.pdf]

cancers

MDPI

Supplementary Materials: Integrative Analysis Reveals Subtype-Specific Regulatory Determinants in Triple Negative Breast Cancer

Shujun Huang, Wayne Xu, Pingzhao Hu and Ted M. Lakowski

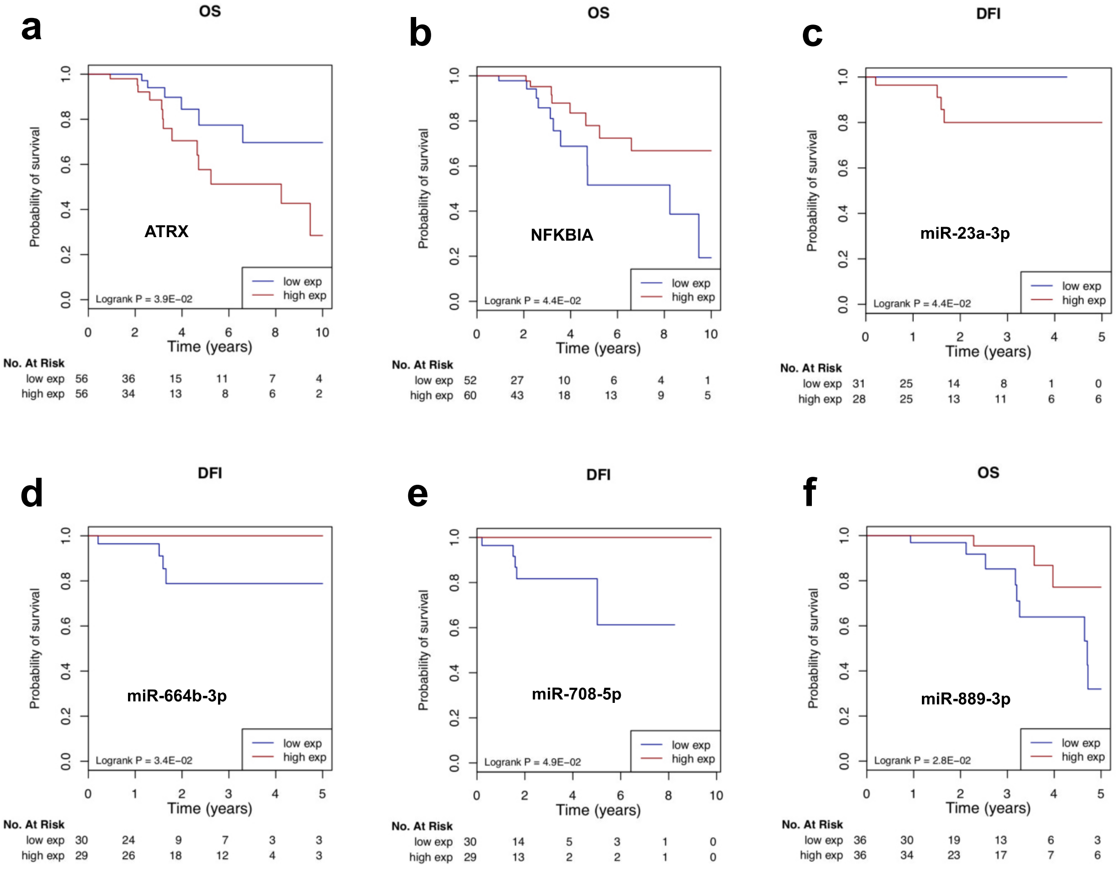

Figure S1. Survival analysis of the TNBC regulators in TNBCs. Based on gene expression levels of a given regulator, TN patients from TCGA were divided into over-expressed ("high-exp", red line) and down-expressed ("low-exp", blue line) groups. Survival analysis with different endpoints (OS, DSS, PFI, DFI) was performed for each regulator. Only significant results were shown. 8 regulators alone could be a prognostic marker for TNBC patients: (**a**) ATRX, (**b**) NFKBIA, (**c**) hsa-miR-23a-3p, (**d**) hsa-miR-664b-3p, (**e**) hsa-miR-708-5p, and (**f**) hsa-miR-889-3p. OS, overall survival; DSS, disease-specific survival; PFI: progression-free interval; DFI, disease-free interval.

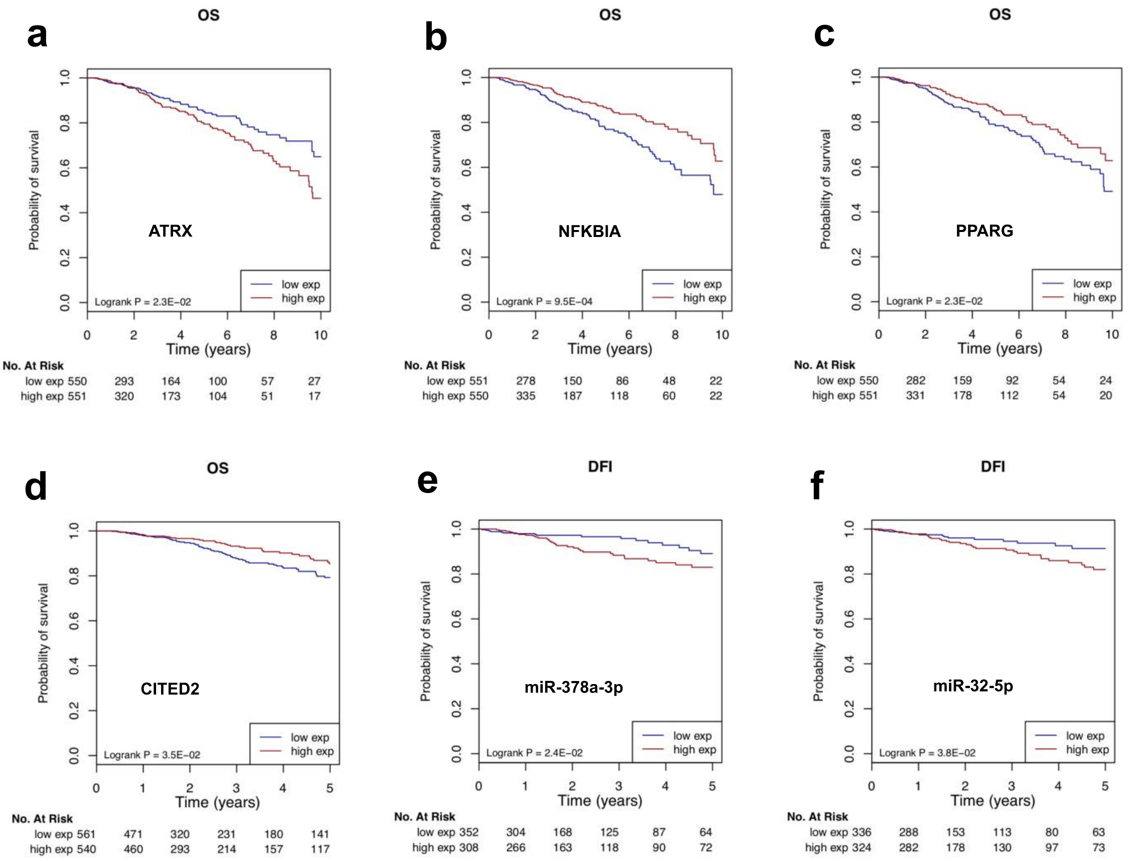

Figure S2-1. Survival analysis of the TNBC regulators in BCs. Based on gene expression levels of a given regulator, BC patients from TCGA were divided into over-expressed ("high-exp", red line) and down-expressed ("low-exp", blue line) groups. Survival analysis with different endpoints (OS, DSS, PFI, DFI) was performed for each regulator. Only significant results were shown. 11 regulators alone could be a prognostic marker for BC patients (six were shown here): (**a**) ATRX, (**b**) NFKBIA, (**c**) PPARG, (**d**) CITED2, (**e**) hsa-miR-378a-3p, and (**f**) hsa-miR-32-5p. OS, overall survival; DSS, disease-specific survival; PFI: progression-free interval; DFI, disease-free interval.

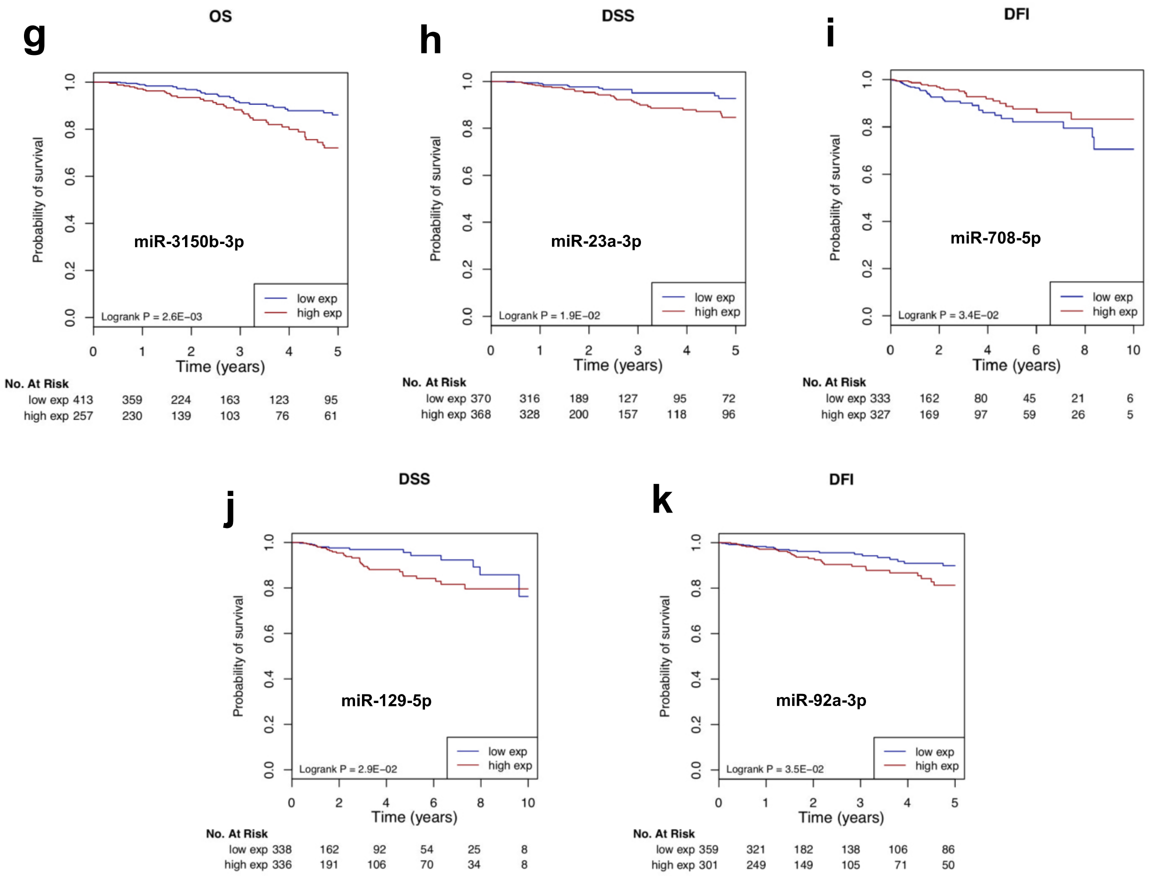

Figure S2-2. Survival analysis of the TNBC regulators in BCs. Based on gene expression levels of a given regulator, BC patients from TCGA were divided into over-expressed (“high-exp”, red line) and down-expressed (“low-exp”, blue line) groups. Survival analysis with different endpoints (OS, DSS, PFI, DFI) was performed for each regulator. Only significant results were shown. 11 regulators alone could be a prognostic marker for BC patients (five were shown here): (**g**) hsa-miR-3150b-3p, (**h**) hsa-miR-23a-3p, (**i**) hsa-miR-708-5p, (**j**) hsa-miR-129-5p, and (**k**) hsa-miR-92a-3p. OS, overall survival; DSS, disease-specific survival; PFI: progression-free interval; DFI, disease-free interval.

© 2019 by the authors. Licensee MDPI, Basel, Switzerland. This article is an open access article distributed under the terms and conditions of the Creative Commons Attribution (CC BY) license (http://creativecommons.org/licenses/by/4.0/).
